# ‘Saving the lives of our dogs’: the development of canine distemper vaccine in interwar Britain

**DOI:** 10.1017/S0007087413000344

**Published:** 2013-07-05

**Authors:** MICHAEL BRESALIER, MICHAEL WORBOYS

**Affiliations:** * Centre for History of Science, Technology and Medicine, South Kensington Campus, Imperial College, London SW7 2AZ, UK. Email: m.bresalier@imperial.ac.uk.; † Centre for the History of Science, Technology and Medicine, Oxford Road, University of Manchester, Manchester M13 9PL, UK. Email: michael.worboys@manchester.ac.uk.

## Abstract

This paper examines the successful campaign in Britain to develop canine distemper vaccine between 1922 and 1933. The campaign mobilized disparate groups around the common cause of using modern science to save the nation's dogs from a deadly disease. Spearheaded by landed patricians associated with the country journal *The Field*, and funded by dog owners and associations, it relied on collaborations with veterinary professionals, government scientists, the Medical Research Council (MRC) and the commercial pharmaceutical house the Burroughs Wellcome Company (BWC). The social organization of the campaign reveals a number of important, yet previously unexplored, features of interwar science and medicine in Britain. It depended on a patronage system that drew upon a large base of influential benefactors and public subscriptions. Coordinated by the *Field* Distemper Fund, this system was characterized by close relationships between landed elites and their social networks with senior science administrators and researchers. Relations between experts and non-experts were crucial, with high levels of public engagement in all aspects of research and vaccine development. At the same time, experimental and commercial research supported under the campaign saw dynamic interactions between animal and human medicine, which shaped the organization of the MRC's research programme and demonstrated the value of close collaboration between veterinary and medical science, with the dog as a shared object and resource. Finally, the campaign made possible the translation of ‘laboratory’ findings into field conditions and commercial products. Rather than a unidirectional process, translation involved negotiations over the very boundaries of the ‘laboratory’ and the ‘field’, and what constituted a viable vaccine. This paper suggests that historians reconsider standard historical accounts of the nature of patronage, the role of animals, and the interests of landed elites in interwar British science and medicine.

On 4 February 1933 *The Field*, England's leading magazine of country sport and life, ran a twelve-page special supplement celebrating the conquest of canine distemper. ‘Saving the lives of our dogs’ told the story of a decade-long effort to develop a preventive vaccine against ‘the scourge of dogdom’ (Figure 1).^1^

**Figure 1.**
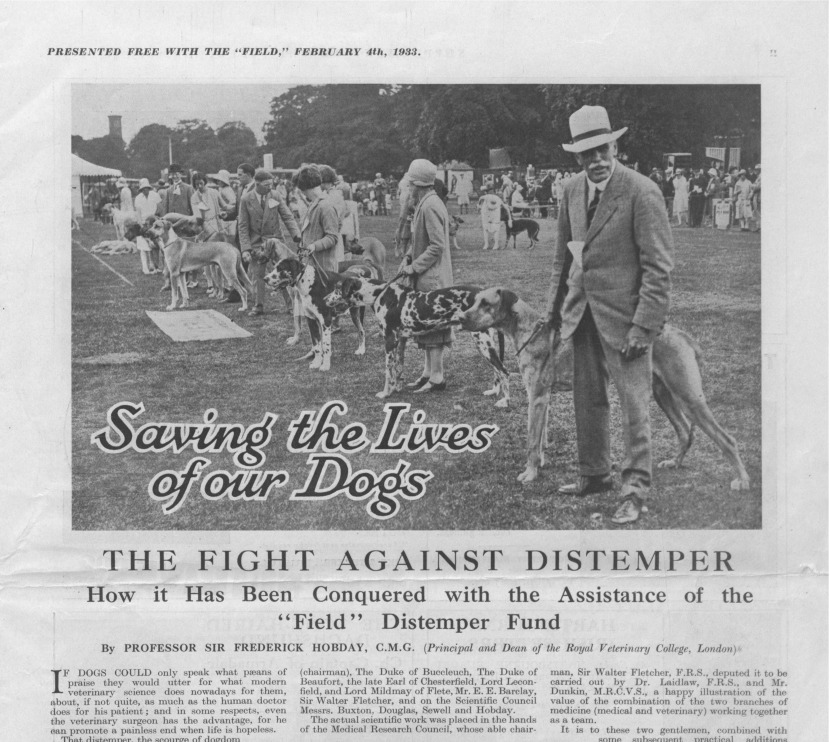
The cover of the special issue of *The Field* celebrating the introduction of dog distemper vaccines. ‘Saving the lives of our dogs’, *The Field*, 4 February 1933, p. 1.

Through the nineteenth and early twentieth centuries, distemper had plagued Britain's dogs and especially ravaged one of the countryside's most cherished symbols and assets – the foxhound. Victor Horsley, surgeon and superintendent of the Brown Animal Sanatory Institute, observed in 1889 that ‘of all diseases, the commonest and the most injurious of the dog is distemper’.^2^ Spurred by this threat, in 1923 *The Field*'s editor, Sir Theodore Cook, established the *Field* Distemper Fund (FDF). With the support of Sir Frederick Hobday, editor of the *Veterinary Journal*, and steered by a select group of landed patricians, the fund raised donations to underwrite an ambitious research programme. Leading microbiological researchers at the Medical Research Council's National Institute for Medical Research (NIMR) in London were engaged to undertake the work. Within a decade success was declared; not only had an effective vaccine been produced, it had also been commercialized, trialled and marketed by the Burroughs Wellcome Company (BWC) at its Wellcome Physiological Research Laboratories (WPRL) at Beckenham in Kent. In the 1930s this success story became iconic, demonstrating of the value of targeted research in state laboratories and public support for such ventures. Historians of interwar science and medicine have overlooked the contemporary scientific importance and public status of this work. In recent histories of the MRC it tends to be discussed as a prologue to research on human influenza or in relation to antivivisection.^3^ Why? We suspect that this is because the distemper campaign concerned animal, not human, health; hence, the epithet ‘a nation of dog lovers’ seems not to extend to British historians of science, technology and medicine.

In this article we retell the distemper vaccine story. Our approach is to follow distemper as it was moved between the field and the laboratory, remade by government scientists, who then worked with veterinarians, the public and scientists in commercial laboratories to develop a range of ‘distemper products’.^4^ Our narrative reveals a number of important, yet previously unexplored, features of interwar science and medicine.^5^ First, the programme was based on a patronage system that relied on a large base of influential benefactors and public subscriptions, reaching across Britain and the empire and into the United States. Historians have chronicled the pattern of patronage involving a single mighty benefactor, such as the Rockefeller Foundation's support of the London School of Hygiene and Tropical Medicine and biomedical laboratories in universities.^6^ The *Field* Distemper Fund was different. It was characterized by close relationships between landed elites and their social networks, veterinarians and their professional bodies, the dog-owning public and its various associations, and senior science administrators and researchers. Second, research supported by the fund saw dynamic interactions between animal and human medicine, which shaped the development of the NIMR's research on virus diseases and established the ferret as a key experimental animal in this area. Finally, the distemper campaign saw the direct translation of laboratory findings into field conditions and commercial products. Rather than a unidirectional process, we show that translation involved continuous negotiations between the constituencies involved over the very boundaries of the ‘laboratory’ and the ‘field’, and what constituted a viable vaccine.

## Distemper before the 1920s

In the first decade of the twentieth century, dog distemper was a disease of national importance and a rallying point for a diverse array of groups. Two groups dominated discussions: landed patricians and veterinarians. The former could rightly claim ownership of the disease, for they had battled it for more than a century.^7^ Seemingly unknown in Britain before the 1790s, distemper developed alongside a new country way of life created around hunting and various sporting pursuits.^8^ The disease attracted special concern because it threatened important symbols of this way of life, the foxhound and the fox hunt. It was also a menace at the new dog shows which became popular from the mid-nineteenth century.^9^ Distemper and how it spread were regularly discussed in the pages of sporting journals, such as *The Field*; in the pedigree breeders’ newspapers, such as the *Fancier's Chronicle* and *Breeders’ Gazette*; and in the magazines of the dog fancy, such as the *Dog's Own Annual*. And it caused suffering and death to family pets, as by this time the dog had been given the new role of ‘companion animal’. William Hunting, founding editor of the *Veterinary Record*, wrote in 1902 of the social costs of the distemper:

The disease causes an annual money loss which we would be afraid to estimate. Money alone does not represent the loss of dog life. ‘The friend of man’ is not valued in coin, and few are the veterinarians who could not tell of the plaintive request from owners to spare no trouble or expense to save the life of a favourite. Without going so far as to say that shooting and hunting are the firmest social bonds of country life, we may safely affirm that sport occupies as much time and gives rise to as much enjoyment as any other function of life in the country.^10^

He went on, ‘Prevention of the loss of dogs due to distemper is, then, a matter of great importance, and one which ought to attract sympathy from a large circle.’

Veterinarians played a key role in developing ways of understanding distemper, which linked to an important transition in the profession.^11^ For much of the nineteenth century, veterinary practice had concentrated on large animals, with the horse being the most important, economically, culturally and professionally.^12^ This changed from the mid-nineteenth century, as the livestock economy grew in importance and pedigree breeding of cattle, sheep, cats and dogs developed. In the case of dogs, the growing fashion for hunting, kennels and pet ownership created a new market for veterinary surgeons.^13^ By the turn of the twentieth century veterinarians were claiming unique expertise over canine distemper and were critical of popular understandings that saw it as a catch-all ailment and of the many proprietary remedies on the market.

While veterinarians saw distemper as a specific disease, it had protean symptoms, making it difficult to diagnose and treat. Infected dogs would first show a fever, vomiting and lethargy, and in a second phase develop weeping eyes and nose, broncho-pneumonia and diarrhoea, which could run into chorea and fits. A widely noted symptom was the hardening of the paws, but this was not a definitive clinical sign. Its variability in presentation was commonly attributed to the environmental conditions in which dogs lived and the constitutional susceptibilities of different breeds.^14^ The large kennels of hunting packs were deemed particularly dangerous because of the concentration of animals in unsanitary conditions, as well as the fact that such animals were ‘higher bred’. Selection for strength with scent, stamina and speed was believed to weaken other bodily attributes.^15^

From the mid-1870s bacteriological work on distemper introduced new aetiological approaches to the disease. British veterinarians had been reluctant to adopt the laboratory-based germ practices of bacteriology of the last quarter of the nineteenth century, though they rapidly adapted germ theories to support their preferred livestock disease control policies of quarantine, disinfection and slaughter.^16^ Once distemper was accepted as a contagious germ disease, the veterinary gaze turned to places and conditions where groups of animals could ‘catch’ it: hunting packs, kennels, dog shows and the street. But establishing the identity of the specific causative agent of distemper proved challenging.

**Figure 2.**
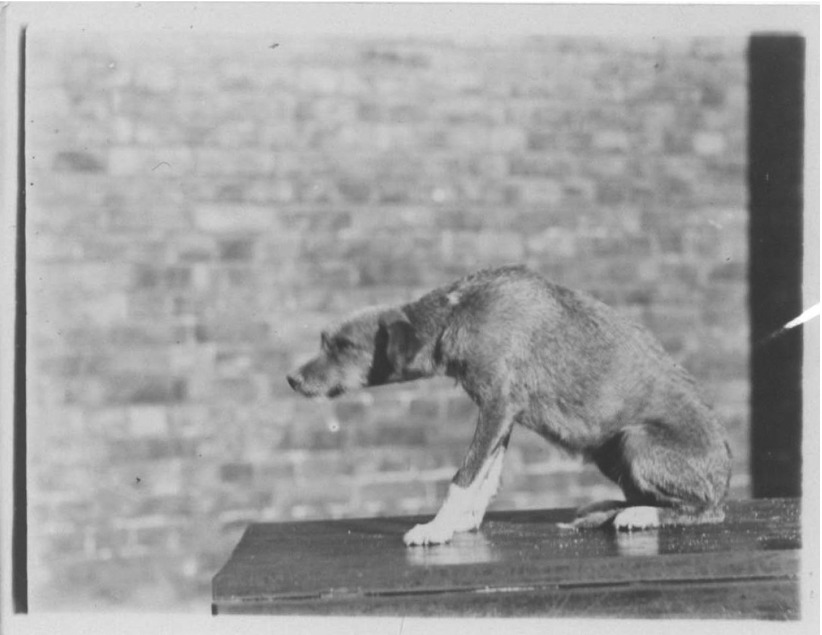
Copeman's Experimental Distemper. ‘Day before death’, the Lister Institute, 1900. Source: National Archives, FD 1/1286.

Investigators from various specialisms in different parts of Europe identified a number of different distemper germs, and, likely inspired by Pasteur's work on rabies, tried to develop a vaccine.^17^ British studies began in 1890 when Everett Millais, eldest son of the pre-Raphaelite painter John Everett Millais, who was a dog breeder and part-time biomedical researcher at C.S. Sherrington's laboratory at St Thomas's Hospital, claimed to have isolated ‘the pathogenic microbe of distemper’ and to have made a protective vaccine.^18^ While the vaccine proved ineffective and discredited the role of Millais's microbe, the announcement spurred other work. The first germ to be given widespread consideration in Britain was isolated by the pathologist and leading smallpox vaccine researcher Sidney Monckton Copeman in 1900.^19^ Convinced that the small bacillus he found in the mouths of dogs was the infecting agent, Copeman used it to produce an ‘experimental distemper’ and a vaccine (Figure 2).

However, there were other candidate microbes and vaccines. In 1901, two French microbiologists, J. Lignières and Charles Phisalix, identified *Pasteurella canis* as the primary cause and soon an apparently effective vaccine, made by the Pasteur Vaccine Company, was available in Britain.^20^ Controversy soon followed this rush of laboratory work. An acrimonious debate between two leading canine specialists, Henry Gray and A.J. Sewell, over Phisalix's vaccine prompted an investigation by a committee of prominent veterinarians in 1903. The committee's investigations resolved little, with the majority deciding that neither Phisalix's nor Copeman's vaccine was an effective prophylactic, implicitly questioning the role of their respective microbes.^21^ In 1905, the French pathologist Henri Carré announced that distemper was caused by a filterable agent, which he had succeeded in experimentally transmitting to dogs, but had failed to isolate in artificial culture or to make visible by light microscopy. Carré's claim was challenged by American and British researchers who aligned themselves behind another new agent, *Bacillus bronchisepticus*.^22^

In Britain, veterinary investigations into controversies surrounding the aetiology of distemper and the value of vaccines raised questions about the role government might play in their resolution. The limited work of the 1903 committee made it clear that the veterinary profession lacked resources and expertise to study the disease. Recognizing this shortcoming, John M'Fadyean, president of the Royal Veterinary College (RVC), joined with a group of influential patricians, headed by the Duke of Beaufort, to propose the creation of a new Committee on Distemper in Dogs under the Board of Agriculture and Fisheries.^23^ Patricians were important in this venture. They had significant social capital at their disposal to get their interests onto government agendas and linked their hereditary privilege with a sense of moral duty to the nation. The Duke of Beaufort chaired a group that included Lord Leconfield, a keen huntsman, who opened Petworth Estate to the public and donated Scafell Pike to the National Trust.^24^ The government refused to fund its work, which led the committee to seek public subscriptions.^25^ In the event, the Board of Agriculture and Fisheries contributed some funds, but little in the way of practical value was produced and the committee was disbanded before the First World War.

The collaborations underpinning the distemper campaign, as well as roles taken by the landed interests, farmers and the veterinary profession, had continuities with other initiatives to develop research on animal diseases. While the control of major livestock diseases was dominated by the state and its agencies, Abigail Woods has recently shown how work on contagious abortion involved a ‘partnership’ between farmers, veterinarians and the state, in which farm associations funded and shaped scientific work and policy on the disease.^26^ Farmers and dairies were enrolled in the long-running investigations into bovine tuberculosis, especially in the work of the Royal Commission that ran from 1901 to 1911.^27^ However, the most direct parallel was the Grouse Disease Inquiry, which began in 1904. A committee of ‘moor owners and grouse shooters’ was constituted by the Board of Agriculture but was required to be self-financing.^28^ Field observations and some laboratory work were undertaken. Landowners, tenants and gamekeepers cooperated with leading biologists and medical scientists, including A.E. Shipley, C. Seligmann, G.S. Graham-Smith and Louis Cobbett. The main field worker was Edward Wilson, who joined Scott's ill-fated Antarctic expedition at the end of his contract.^29^ A review of the final report in *Nature* observed that the initiative was ‘a striking, and we believe, a unique example of what can be done by the combined efforts of sportsmen, gamekeepers, field-observers, and biological experts’.^30^

## The *Field* Distemper Fund

In the aftermath of the war, distemper returned as a galvanizing issue among country elites and leading veterinarians. Yet the prospects for controlling the disease were bleak; neither preventive nor curative measures had altered high mortality rates. Suggestions to follow livestock stamping-out policies with hunt packs and putting down individual dogs were rejected by owners and threatened relations with veterinarians’ valuable clients.^31^ Veterinarians argued that new work on distemper's aetiology was desperately needed, and they looked to laboratory science for solutions.^32^ Bacteriology in particular had become a crucial part of the modernizing ambitions of the profession.^33^ Frederick Hobday, who was a leading advocate of this modernizing mission, argued that veterinary expertise had to be reorganized around developing and employing effective measures of disease prevention, made in the laboratory and deployed in the field.^34^ One early result was the creation in 1917 of the Central Veterinary Laboratory at Weybridge and laboratories for animal pathology at Cambridge and Edinburgh.^35^

The campaign against distemper carried these aspirations forward. It began in autumn 1922 when Sir Theodore Cook, editor of *The Field*, and a group of powerful patricians launched the *Field* Distemper Fund. Noting the ‘futility of commencing research on any small or indefinite sum’, as had been the case in previous efforts, the fund's council set a fundraising target of £25,000 (equivalent to £4.2 million in 2010).^36^

The fund reprised pre-war funding models, this time with the MRC as partner, thus ensuring the central role of government science. Cook approached the council's secretary, Walter Morley Fletcher, in October 1922 with a proposal for cooperation on the ‘Distemper Question’.^37^ Cook and his colleagues were convinced that new research would be best served by a ‘centralising effort’, overseen by a single scientific body.^38^ The MRC had positioned itself as such an authority.^39^ It had made experimental work with animals central to its programme. The value of this focus was being shown by investigations it coordinated into vitamins, hormones and, beginning in the same year as the distemper appeal, insulin.^40^ Dogs played crucial roles in the elaboration of the research on all these topics.^41^ Crucially for the FDF, the MRC was deeply invested in the ideology of translating knowledge and tools produced in experimental animals into clinically applicable knowledge and practice.

Cook's proposal was also attractive to the MRC on practical grounds. As part of its mandate, and due to strict restrictions on the amount of government funding it received, the MRC actively sought non-government sources of support. Before Cook approached Fletcher, the MRC had already been making plans to investigate distemper as part of a new research programme on diseases suspected as being caused by so-called ‘filterable viruses’. Impetus for this programme came from preliminary investigations into the role of a filterable virus in the 1918–1919 influenza pandemic.^42^ Fletcher stressed the relevance of distemper in his annual report for 1921–1922:

There is good reason to think that [dog distemper] offers a close parallel to human influenza. It seems probable that the infective agent is a filterable virus, and that here also the severity of the resulting disease depends largely upon secondary infections, facilitated by the primary infection. There is ground for hope that the study of dog's distemper under strict experimental conditions may throw important light upon analogous problems of human disease, and at least suggest new clues for investigation or new technical methods for the investigator. It is with the primary object of gaining knowledge of human disease that the Council decided to support further study of distemper in dogs. On that ground alone they find complete justification of the expenditure of part of their funds in this direction.^43^

FDF patronage made it possible for the MRC to realize Fletcher's ambition. He noted in a letter to Cook in November 1922 that, ‘even if there is no parallel in human beings to distemper in dogs, the investigation into the latter might well prove to give some valuable clue – to guide in studying one or more of the numerous virus diseases in human beings’.^44^

FDF support also represented an opportunity for the MRC to establish itself in animal disease research. Fletcher had been trying to stake out territory in this area, guided by the view that veterinarians, like clinicians, were unequipped to pursue laboratory-based research.^45^ He and his colleagues repeatedly clashed with leading veterinarians over the development and control of veterinary science.^46^

**Figure 3.**
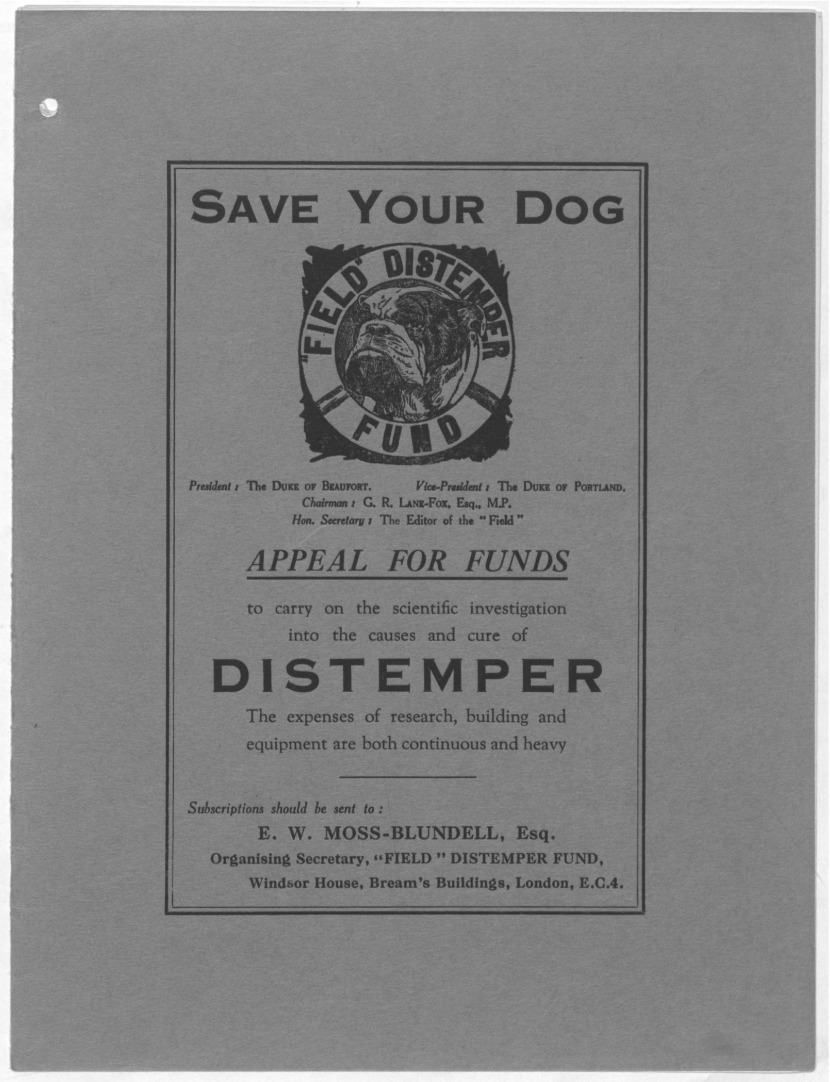
The *Field* Distemper Fund Appeal. Advertisement as part of fundraising campaign. *The Field*, February 1925.

Historians have traced the patronage relations that Fletcher established with single, large donors, such as the Rockefeller Foundation and Dunn Trust, to construct new disciplinary foundations in fields of physiology, chemistry and pathology.^47^ The MRC's collaboration with the *Field* Distemper Fund relied upon a different system. The fund was built through soliciting voluntary contributions directly from small organizations and individuals (Figure 3). Cook made appeals through the pages of *The Field*, and was helped considerably when the *Daily Telegraph* did the same.

These efforts drew upon hunting, kennel and breeding networks that stretched across Britain and the empire – even to Canada, Australia and India – and, most significantly, into the United States. When detailed figures were first reported to the end of 1925, the fund had raised nearly £16,720 (£2.48 million in 2010), from 1,700 donations from individuals and societies.^48^

The *Field* Distemper Council (FDC) oversaw fund-raising and publicity, while a Distemper Research Committee (DRC) was established ‘to initiate and direct the scientific work’. Parallel research in the United States was organized by an American Distemper Committee, which had representatives on the British *Field* council.^49^ Regular progress reports were sent to major subscribers and published in *The Field*, veterinary journals and the general press, creating a considerable constituency of, to use the modern jargon, ‘stakeholders’ in the research.^50^

**Figure 4.**
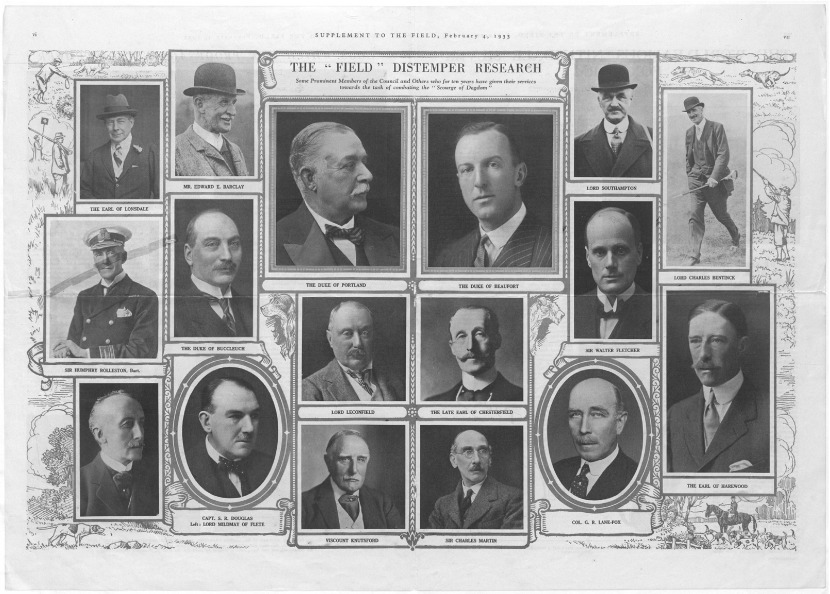
The *Field* Distemper Research Council, *The Field*, 4 February 1933, p. 9.

Landed patricians and those closely allied to their interests dominated the FDC (Figure 4). Its president was the 9th Duke of Beaufort, Henry Hugh Arthur Fitzroy, an Etonian, founder of the Badminton horse trials, and ‘sportsman by profession’. Chairman of the Masters of Foxhounds Association, which was founded by his father, he hunted his own pack on the family estate in Somerset and knew every aspect of the sport, from kennel management to the correct position of a tiepin. Other lay members had similar pedigrees.^51^ The scientific members were leading lights of British medical science, and they controlled the DRC, which was chaired by Charles Martin, professor of pathology and director of the Lister Institute.^52^ Fletcher had a foot in both camps, being an avid hunter and outdoorsman who maintained a country home and close relations with the landed gentry.^53^ Bringing together representatives from both animal and human pathology, the DRC aimed to establish ‘a constant and invaluable interchange of ideas and methods’.

Between 1923 and 1932, £55,000 was spent on distemper research at the NIMR, equivalent to £8.8 million in 2010.^54^ The largest proportion, £22,000, came from voluntary donations from Britain and the empire, with a further £15,000 from the American Distemper Committee and the remaining £18,000 from the MRC's grant-in-aid.^55^ In Britain large sums came from the *Daily Telegraph* Fund, the Kennel Club, Spratt's Patent, Spillers, the Ladies Kennel Association and the Masters of Foxhounds Association. The empire contributed, especially India, where the Maharajahs of Jind and Patiala, the Ootacamund Hunt, and the Madras Hunt all made generous donations. In total, over 3,500 organizations and individuals made donations to the fund. Mostly drawn from the upper and middle classes, their contributions gave them a direct stake in the project.^56^ As we will see, the DRC and the researchers at the NIMR enrolled these new stakeholders into the actual investigations, asking them to volunteer their dogs for experimental fieldwork; in turn they were able to make demands on the process and products of the investigations.

## Distemper and the research laboratory

The fulcrum for the MRC's distemper research was the NIMR's Department of Bacteriology and Experimental Pathology, led by the Cambridge-trained biochemist and experimental pathologist Patrick Laidlaw. He had previously worked with the NIMR's acting director Henry Dale on histamine at the WPRL, which in 1921 had moved from Brockwell in South London to Langley Park in Kent to accommodate more animal work.^57^ Laidlaw was recruited in 1922 to develop the virus programme, and quickly made distemper his primary focus, working with G.W. Dunkin, a veterinary pathologist and superintendent of the NIMR's animal facilities.^58^ They started from the assumption that a key problem in previous experimental investigations of distemper had been not having ‘standard’ animals that were guaranteed to be free from prior infection.^59^ Fletcher and Dale devised a large-scale, centralized animal-breeding programme that was to be geographically separate from the NIMR.^60^ In 1921, the MRC purchased a forty-acre agricultural site at Rhodes Farm at Mill Hill. This relocation and the provision of animal facilities were aided immensely by monies from the Distemper Fund.^61^ The ‘Farm Laboratories’ had provision for the breeding and housing of dogs and other large animals, a well-equipped laboratory and an isolation compound for quarantining dogs with distemper.^62^

**Figure 5.**
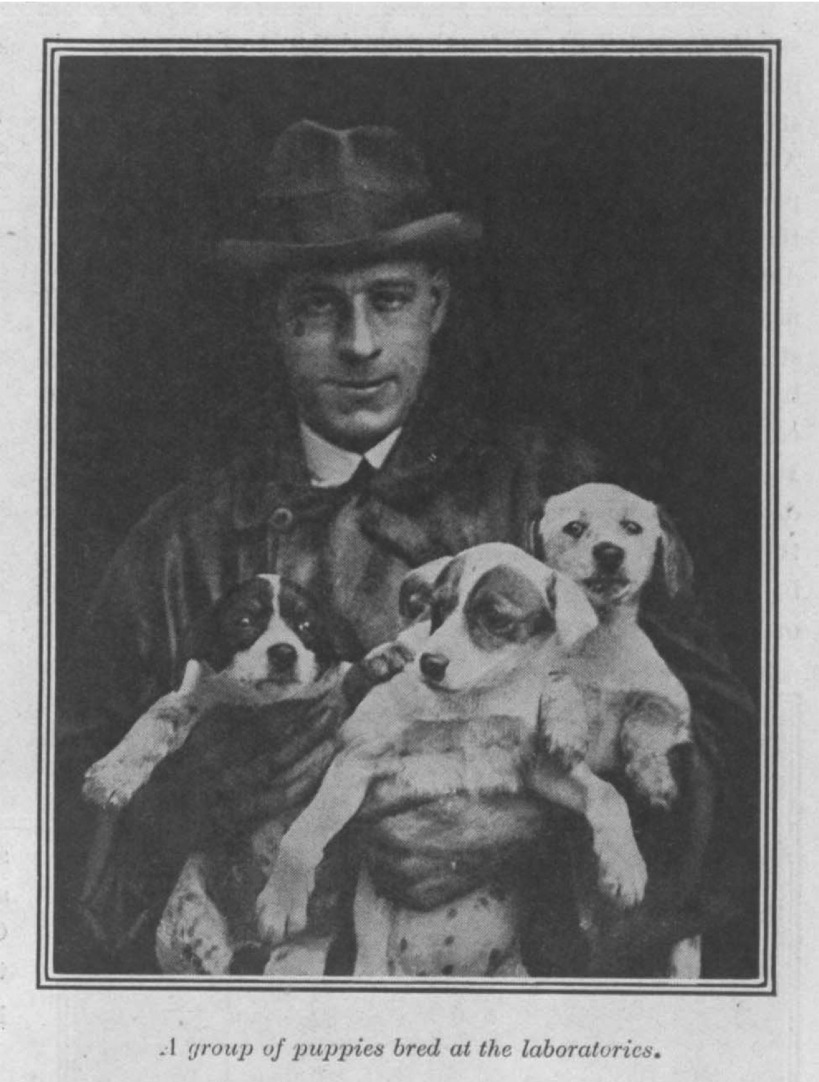
G.W. Dunkin with purpose-bred distemper research puppies. The *Field* Distemper Fund Report for 1925, p. 8. Source: National Archives, FD 1/1274.

Starting in the summer of 1923, Dunkin began to create purpose-bred dogs for experimental work on distemper. To establish the stock, he and Laidlaw decided to use ‘bitches of any sort of breed’.^63^ Considerations of pedigree were unimportant to Dunkin and Laidlaw in their research, as they believed that all dogs were equally susceptible to distemper and that work done on ‘cross-breds’ would give their results wider public acceptance. Using older dogs to start the breeding programme was important because these were likely to have had distemper and thus be immune. In the first one and a half years, 110 purpose-bred puppies were reared at Mill Hill, creating a stock that became the basis for constructing a new experimental distemper (Figure 5).

The first aim of Laidlaw and Dunkin was to settle the dispute over the causative agent of the disease.^64^ The core issues were technical and methodological, with neither side being able to sufficiently control the susceptibility of dogs to experimental infection.^65^ With their purpose-bred dogs, Laidlaw and Dunkin could control this variable. In their first experiments, they began by putting a healthy puppy in contact with dogs experimentally infected with distemper, and once the puppy contracted the disease, they tried to transmit it to other healthy puppies. While they were able to do so, they were unable to produce cultures of known bacilli associated with distemper from material that induced the experimental infection.^66^ Their conclusion was that this result ruled out a primary role for a bacterium, including *B. bronchisepticus*, and that the filterable virus identified by Carré was a more likely candidate.

Carré had relied upon filtration to produce a bacteria-free inoculum that was infective for dogs. However, his methods proved difficult to replicate. Laidlaw and Dunkin chalked this up to two constraints: (i) the highly variable symptoms and severity of distemper in dogs, and (ii) the lack of a reliable source of pathogenic material for laboratory work. Their purpose-bred, infection-free puppies removed both obstacles. Their strategy was to stabilize and standardize the disease in the dog, which would allow them to study and manipulate the virus *in vivo*. First, they produced and characterized a new disease entity – ‘experimental dog-distemper’ – with a typical clinical picture: it was an acute infection, with an incubation period of four days, followed by fever, discharge and severe gastro-intestinal symptoms. Crucially, it was marked by ‘an unusual temperature curve’, where the onset of febrile symptoms could be associated with the presence of the pathogen. Experimental distemper rarely killed the dogs, but it was easy to transmit serially and, just as important, it could be used to provide reliable pathogenic material.

Previous researchers had identified blood, sera and various organs, including the brain, as highly infective, but Laidlaw and Dunkin found that the best material came from the liver, spleen and mesenteric glands extracted post-mortem.^67^ Preparing bacteria-free filtrates of these materials was also important for countering a common criticism of Carré's inoculums, namely that they still contained *B. bronchisepticus.*^68^ Filtration was one of the most technically challenging areas of virus work and, to address this issue, Laidlaw and Dunkin turned to their NIMR colleagues J.E. Barnard and W.J. Elford, who contributed physical and biochemical expertise to improve techniques for filtering, purifying and measuring ‘ultramicroscopic’ agents from blood and tissue.^69^ By early 1926, Laidlaw and Dunkin claimed that ‘the infecting agent of dog-distemper belongs to the class of filter-passing viruses’ on three counts: its filterability, its resistance to cultivation, and its dependence on living tissue such that it could not be grown ‘outside the [animal] body’.^70^ They named the new agent ‘Rhodes virus’, after the farm at Mill Hill, and it became their master strain.^71^

For the first year and a half of their research, Laidlaw and Dunkin worked solely with dogs, but these were not ideal subjects. As Tilli Tansey has shown, the NIMR's use of dogs was the focus of vociferous anti-vivisection agitation around the proposed Dogs’ Protection Bill.^72^ Dogs were also expensive to keep, did not breed rapidly and were emotionally unsuited to strict isolation quarantines.^73^ In late 1924 Laidlaw and Dunkin introduced the ferret as a new experimental animal.^74^ At the suggestion of S.R. Douglas, director of the Institute's Department of Bacteriology and Experimental Pathology and through their connections with the *Field* Distemper Council, they learnt that ferret handlers reported that the animal was highly susceptible to canine distemper.^75^ In Britain, ferrets were used for rat control and rabbit hunting, and in the working-class ‘sport’ of ferret-legging. Domestication into the NIMR's virus programme was the first time the ferret was used for medical research and the start of a long scientific career.^76^

**Figure 6.**
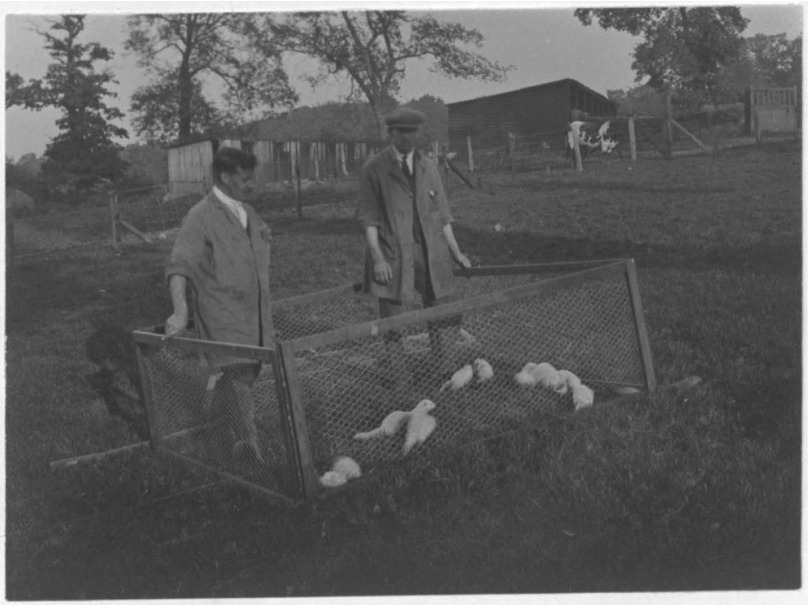
Purpose-bred ferrets at Mill Hill. Date unknown (*c.*1924–1926). Source: National Archives, FD 1/1284.

The ferret quickly became Laidlaw and Dunkin's preferred research animal, because distemper was easy to reproduce and to identify – it was invariably fatal (Figure 6). Also, the virus tended to be concentrated in the spleen, which provided a ready source of experimental material. All their ferrets were purpose-bred at Mill Hill in a special hut isolated from the rest of the facility, and they bred readily and quickly; producing up to three hundred kits (young) per year.^77^ Moreover, unlike the dog, they were known to thrive in small spaces, which made them well suited for confinement in laboratory cages. Contrary to their reputation as vicious predators, Laidlaw and Dunkin found ferrets easy to manage and they became invaluable partners in the research.^78^

In their experimental system, Laidlaw and Dunkin initially ran every important research line on both the dog and the ferret, but the latter was chosen for the development of an experimental vaccine. Attempts at vaccination had been made in Italy, France and Germany, but without purpose-bred animals. Laidlaw and Dunkin reviewed and largely dismissed this early work.^79^ Although they were confident they now had the ‘right’ pathogen, along with the facilities and techniques for its manipulation, they found, as others had before them, that producing virus vaccines was difficult.^80^ The key obstacle was the lack of artificial culture media and techniques with which to purify the pathogen. The problem was repeatedly highlighted in MRC and DRC reports, and in the medical press.^81^ Virus vaccines had to be produced and standardized at the whole-animal or tissue level, as in the well-established vaccines used for smallpox and rabies.^82^ In the 1920s, researchers searched for improved methods for purifying viruses and producing virus vaccines, and NIMR's work on dog distemper was at the forefront of this research programme in Britain and internationally.

The goal of vaccine development was to produce standard, high-quality antigens that, when inoculated, would induce artificial immunity by stimulating antibody production.^83^ An ideal antigen generated immunity without causing disease. There were two types of vaccine: ‘live’ and ‘killed’. The former involved using an attenuated (weakened) virus to produce a sub-clinical or mild infection, while the latter used dead virus particles with antigenic properties. Killed virus vaccines were made by either heating the virus or treating it with chemicals. Laidlaw and Dunkin chose a killed vaccine, treating their spleen extract with formalin, a method they borrowed from researchers working on a foot-and-mouth vaccine at the Lister Institute.^84^

After trialling various protocols, Laidlaw and Dunkin found that the most effective was a two-step process.^85^ It involved, first, administering the formolized vaccine to stimulate antibody production, and then fourteen days later injecting a dose of the live virus, to reinforce the immune response, and make it longer-lasting.^86^ They refined their system in trials in ferrets that ran through 1927 and demonstrated that it was safe and effective in both laboratory and field conditions; for the latter, they worked with three ferret keepers from country estates.^87^ However, when the ferret vaccine was trialled with dogs, it provided only limited protection.^88^ Laidlaw and Dunkin soon determined that an effective dog vaccine required a ‘homologous’ antigen made from dogs.^89^ This meant developing methods to prepare a virus that was produced and extracted from dog tissue, as explained in the *Veterinary Record* early in 1928:

A dog … was taken when it was exceedingly ill with distemper. The dog was killed; the spleen, mesenteric glands and liver were taken out, put through a sausage machine and ground up in a mortar. The resultant product was mixed with saline and shaken vigorously. The connective tissue fibres were strained off through a double thickness of muslin. Enough formalin was added to give a strength of 1–1000, which killed the virus.^90^

While dogs were used as sources of the virus for vaccine production, ferrets continued to be used as the source of the live virus for the second inoculation.

Vaccine development for dogs moved rapidly from the laboratory to the field. Trials began in 1928 and were coordinated through the FDC, whose membership was used to enrol masters of foxhounds, veterinary surgeons and dog owners.^91^ Laidlaw and Dunkin oversaw the selection of participants and controlled the provision and testing of the vaccine. Unusually, they had special permission, under the animal experimentation legislation, to perform experiments when they visited estates, kennels and homes.^92^ They insisted that only veterinary surgeons administer the vaccine and the virus, and only according to specific guidelines. The trials compared vaccinated with unvaccinated animals, using a rather ad hoc notion of control.^93^ The trial design relied on both groups of animals being ‘naturally’ exposed to distemper, which was thought to be especially rife in countryside. Packs of foxhounds, as well as breeds from established kennels, were the main trials subjects. Leading hunts were keen to volunteer their dogs and to participate in the studies. Foxhounds were useful because of their breeding and maintenance, while other dogs were studied to determine the range of protection. In the first round, 340 foxhounds and a hundred other dogs were vaccinated, with only two deaths.^94^ The results were compelling and the trial was extended, so that by November 1928, two thousand dogs had been vaccinated.^95^ Only a small percentage of vaccinated dogs (an estimated 1 per cent) contracted distemper, compared to infection rates of between 50 and 75 per cent in unvaccinated dogs. These results, much more than the earlier laboratory demonstrations, confirmed for veterinarians, dog owners and many scientists that the distemper characterized by Laidlaw and Dunkin in the laboratory was truly a virus disease in the field.^96^

On receiving Laidlaw and Dunkin's report on the trials in November 1928, the FDC quickly released a summary to the veterinary and general newspaper press, generating a wave of interest and enthusiasm, assessments of the practical implications of the vaccine, and immediate demands for its general release.^97^ The success was celebrated in national and local newspapers, Laidlaw and Dunkin were front-page celebrities in the *Daily Mirror*, and the *Manchester Guardian* observed, ‘There should a concerted wagging of tails throughout the world's kennels at the good news for dogdom that has been announced.’^98^ Laidlaw and Dunkin received all the plaudits and this irritated other participants in the campaign. The FDC also wanted recognition, especially for the role of Sir Theodore Cook, who had died earlier in the year, just before the trials. The *Veterinary Journal* suggested that

there can be no doubt that had it not been for [his] practical encouragement and generous help … together with the clever propaganda appeals issued by the persevering Secretary of the Fund … the money would not have been forthcoming, and this Research could not have been done.^99^

Veterinarians were also quick to claim credit, particularly for the crucial transition from the laboratory to the field. In the early stages of the research, when Laidlaw and Dunkin were constructing their ‘experimental distemper’, veterinarians validated the correspondence between the disease at the NIMR and that met with in their practices. Similarly, field trials of the vaccine depended on the ability of veterinarians to carry out inoculations, report results, identify cases of distemper and suggest modifications to the procedure. They also maintained contacts with kennel masters and dog owners. The *Veterinary Record* grasped the moment ‘to emphasise the great advantages that must necessarily follow collaboration and discussion between the research workers and clinicians’ and that ‘open and free discussion of the knotty problems as they occur … conduces towards that harmonious working which is absolutely essential to complete success’.^100^ The harmonious relations between the laboratory and the field that the *Veterinary Record* welcomed and looked forward to continuing became considerably strained in the process of translating Laidlaw and Dunkin's experimental vaccine–virus into a commercial product.

## Distemper and the commercial laboratory

Encouraged by the early success of the field trials, the DRC pushed for the commercial production of the vaccine–virus by companies in Britain and the United States.^101^ Laidlaw and Dunkin were very positive about the development and were keen to hand over their invention, as they were tiring of requests from dog owners and veterinarians for information and their vaccine. Wide public patronage brought expectations of engagement with the research and entitlement to its results. Some people argued that the vaccine–virus should be distributed free to those who had subscribed, and even that it should be made available to all dog owners with a view to eradicating the disease as Jenner's vaccine was promising for smallpox.^102^ Peter Bowler has recently pointed to the high level of ‘public engagement’ by scientists in interwar Britain, detailing the activities of key popularizers, such as Julian Huxley and J.B.S. Haldane, and the number of popular-science magazines.^103^ The distemper research at the NIMR took a different form: rather than just experts engaging the public, a large number of stakeholders were in dialogue and exchange with the researchers and the research process. This dynamic continued in the commercialization of the vaccine–virus.

By 1928, Laidlaw and Dunkin's scientific publications detailed the whole process of manufacturing the vaccine. The MRC and FDC were worried about the ‘marketing of inferior preparations’, so they attempted to control the selection of companies for commercialization.^104^ In Britain, WPRL, where Laidlaw had worked and which had good relations with the NIMR, was approached in early December 1928 and agreed to take on the business.^105^ The vaccine and the science behind it were a ‘gift’ to the company; there was no licence agreement and no payment. The ethos of those leading the FDC saw it as a gift to the nation, and the MRC had to keep its distance from commercial matters as it was a regulatory agency for biological products. To ensure that WPRL maintained control over the commercial vaccine, the MRC agreed that Laidlaw and Dunkin would ‘give every assistance in technical questions of the preparation and standardisation of the product’.^106^ Accordingly, the NIMR researchers advised WPRL's leading veterinary scientist, Thomas Dalling, who worked at their new out-of-town facility at Langley Park.^107^ The American Distemper Committee made similar arrangements with two firms, Lederle Laboratories and Mulford Laboratories, both of which sent representatives to the NIMR to learn the methods of preparation.^108^

In Britain, the move towards commercialization raised issues of ownership of the vaccine.^109^ The treasurer of the Distemper Fund, Lord Mildmay, insisted that the vaccine be named after the fund, thus putting its proprietary stamp on the product it had helped to create and was giving to the nation. R.A. O'Brien, the head of the WPRL, who negotiated the commercial contract with the MRC, rejected the suggestion outright, arguing that there was an important distinction between funding work on an experimental vaccine and funding its commercial manufacture. He argued that BWC's investment in the latter gave the company sole proprietary rights. The MRC agreed, in part because maintaining the distinction was crucial to its own role in the regulation of therapeutic substances. After heated exchanges, an agreement was struck whereby the vaccine was marketed as the ‘Wellcome’ Canine Distemper Prophylactic, with mention of the Fund in its marketing.

In the event, WPRL issued seven distemper prophylactics in the period from January 1929 to May 1931, as set out in Table 1. The table shows that there was no simple transfer of a single Laidlaw and Dunkin invention to the market via BWC; rather it was modified and other products developed. The original vaccine–virus was only commercially available for sixteen months before being withdrawn. Over the following ten months three new experimental products were trialled, before two of these, a vaccine–dry virus and serum–virus, were put on the market in May 1931. The success story of November 1928 and the expected benefits to dogdom became a technical and social struggle to find a distemper vaccine that worked in the field and was acceptable to the dog-owning public at all social levels.

**Table 1.** Issue of Canine Distemper Prophylactics from WPRL, Beckenham. Source: National Archives, FD1/1296, C385909.

1	Experimental vaccine and wet virus	January–April 1929
2	Commercial vaccine and wet virus	April 1929–July 1929
3	Experimental double vaccine	July 1930–January 1931
4	Experimental vaccine and dry virus	January 1931–May 1931
5	Experimental serum and virus	January 1931–May 1931
6	Commercial vaccine and dry virus	May 1931–
7	Commercial serum and dry virus	May 1931–

At the start everything looked very promising. In January 1929, within a month of beginning work, the WPRL, having successfully scaled up Mill Hill methods, issued free ‘experimental vaccine and virus’ to selected veterinarians to use with packs of hounds. Following positive feedback, BWC began commercial sales in late March.^110^ In both phases, Dalling engaged in a large correspondence with veterinarians and others, and promoted the product through talks to branches of the National Veterinary Medical Association (NVMA).^111^ By the end of October, Dalling reported that twelve thousand animals had been successfully vaccinated and in 99 per cent of cases no problems were reported.^112^ Over the months the number of adverse reports, although relatively low at less than 1 percent overall, had totalled over three hundred and included 114 deaths.^113^ Staff at the WPRL and most veterinarians regarded this number of ‘complications’ as acceptable for a new biological product, but the anger of owners whose dogs had suffered or died became a lightning rod for criticism. Those making most noise were amongst the powerful and influential. In December 1929, Lady Burton of Walpole House reported in *Horse and Hound* that a Cairn terrier and two Keeshonds, who had been inoculated in July, had become ill with what her veterinary surgeon diagnosed as ‘distemper’, while another ‘Burton’ puppy was reported as having ‘several hysterical fits’ after being inoculated.^114^ In another exchange, the Duke of Portland reported to Fletcher of vaccine ‘failures’ at the kennel of Lady Howe, president of the British Labrador Club.^115^ Masters of hounds reported that the vaccine–virus caused fits and other nervous conditions, and that it failed to protect against infection. Indeed, the second inoculation with live virus was said often to cause distemper. The very networks that had facilitated distemper research and development were biting back.

Some of these complaints drew on wider anti-vaccination sentiment, which in the late 1920s focused on post-vaccinal encephalitis and led to the introduction of new protocols for smallpox vaccination.^116^ But, more importantly, they also drew on past experience of owners and veterinarians with the many bacterial vaccines and patent medicines for distemper. In a public address to the Central Division of the NVMA, A.A. Comerford, a leading veterinary surgeon who was among the first to use Laidlaw and Dunkin's vaccine, noted that ‘the majority of dog-breeders, remembering the inefficacy of previous attempts at prophylaxis by inoculation, would far sooner run the risk of an outbreak of distemper in their kennels than submit their dogs to inoculation’.^117^ Worries about vaccine-damaged dogs led BWC to hire an agency to collect press cuttings on the emerging epizootic of canine hysteria, which some dog owners blamed on distemper vaccine–virus.^118^

In December 1929, R.A. O'Brien, prompted by the FDC and the Dog Owners’ Club, set up an internal inquiry to find a ‘way out’ of the mounting criticism.^119^ Several likely causes of the problems were identified: the quality of the vaccine, the quality of the virus, the condition of the dogs being vaccinated, young dogs being inappropriately vaccinated, veterinarians’ poor technique with inoculation, and their misdiagnosing ‘complications’.^120^ Dalling went out to NVMA meetings across the country to explain the findings. Supporters noted that a 98–99 per cent success rate was exceptional for any preventive vaccine.^121^ Laidlaw and Dunkin also entered the discussions and argued that most likely the failures were due to processing at WPRL, and veterinarians and owners not following protocols.^122^ Dalling and O'Brien placed some blame on the NIMR workers, doubting the accuracy of the results from their 1928 field trials.^123^ In particular, they questioned the purity, stability and strength of the NIMR workers’ virus preparation. O'Brien even suggested that the success of the NIMR's trial was due to dogs’ previous exposure to wild distemper virus, rather than to the Mill Hill vaccine–virus.^124^

However, privately O'Brien acknowledged that the company had rushed to market too quickly.^125^ For him, the most serious problem was the quality of the virus, which was something they could improve with methods to improve standardization and stability. Laidlaw and Dunkin had prepared the virus in liquid form and, though their production process had been readily scaled up, it was expensive and WPRL found it difficult to match the supply of a product that lost potency over time with demand. The company's first move was to put a forty-eight-hour ‘use-by’ stipulation on each phial and only to send out the virus from Monday to Wednesday, to avoid weekend postal delays.^126^ As well as dealing with production issues, Dalling also experimented with other methods – making a frozen virus, with poor results, and a ‘dry’ virus, that was much more promising.

Meanwhile, complaints about what Dalling was now terming the vaccine–wet virus continued. Through spring and summer 1930, Dalling faced increasingly heated criticisms from veterinarians.^127^ To defuse the issue, BWC withdrew the vaccine–wet virus in June 1930.^128^ O'Brien was blunt in his assessment of ‘our obvious failure to do all this work a year ago’, and repeated that ‘we took too many things for granted’.^129^ He argued that, in addition to the production problems and ensuring that vaccine–wet virus worked in variable field conditions, the company's staff had also suffered from ‘the original research not being our own’, suggesting either that the nuances of the process had not been passed on fully by Laidlaw and Dunkin, or that company staff had assumed too much.^130^

But BWC had already made a considerable investment and were not about to abandon the new market. On withdrawal of the vaccine–wet virus, WPRL had an alternative available: a two-dose vaccine using killed virus, given at a fourteen-day interval. It was offered free to veterinarians from July 1930 for an experimental field trial and said to provide short-term protection, which was said to be better than nothing. In the field, veterinarians found problems securing the timing of the doses and the number of failures led the company to withdraw the product in January 1931. In the same month WPRL began experimental trials with two further products: a vaccine–dry virus and a single, simultaneous inoculation of antiserum and wet virus. Dalling had spoken on the former in May 1931, stating that he had freeze-dried the virus by removing moisture with liquid nitrogen, and reported success with over a thousand doses, some dispatched as far as South Africa and New Zealand.^131^ He claimed that the dry virus was more stable and more easily standardized.

The antiserum-and-virus product was based on different principles. Antisera introduced into the body antitoxins or antibodies against a particular disease harvested from another animal. They typically conferred short-term, ‘passive’ immunity, in contrast with the long-term ‘active’ immunity given by vaccines. Simultaneous inoculation of the live, wet virus and an antidote aimed to keep the dog infection-free, while allowing immunity against the virus to develop. Laidlaw and Dunkin had started work on distemper antisera in 1928, but did not publish details until March 1931.^132^ They reported testing three brands of antisera on the market and finding all of low potency, and then having had success in producing their own high-potency ‘hyperimmune’ serum. They went on to report that, when used with live, wet virus, their antiserum protected dogs in field trials and could be used also to treat dogs already infected. The results were presented ahead of publication to the NVMA in February 1931, where the work was roundly applauded as providing a viable alternative to the virus vaccine.^133^

Already, in January 1931, Dalling had begun issuing combined antiserum and wet vaccine for veterinarians to trial, along with antiserum for use on its own in treatment. As with the earlier products, the NIMR and WPRL field trials with veterinarians and masters of hounds were crucial to establishing the credibility of the product.^134^ There were still many failures, most of which were put down to dogs harbouring infection prior to vaccination. Veterinary expertise in identifying distemper and in administering the products was again deemed necessary to avoid such failures.^135^ Although the antiserum was hard to make and standardize, feedback on its use as a curative was positive and, along with the vaccine–dry virus, it was launched onto the market in May 1931.^136^

In the spring of 1932, Dalling spoke again to the Central Division of the NVMA.^137^ He opened with an apology, stating that ‘there is little new to tell you’. He confirmed that the vaccine–dry virus method was at least 99 per cent effective and had become very popular with veterinarians, perhaps too popular as they were neglecting other precautions. The antiserum–dry virus method had also proved popular because of its convenience, but it had been less successful than hoped. Indeed, failure rates of over 5 per cent had been reported. These had been taken up in the popular press, which still followed the great dog-distemper success story. Dalling worried that the public often did not distinguish between the different methods of preventing distemper, such that ‘every failure rebounds to the discredit of the Laidlaw–Dunkin first method’.^138^ Indeed, he suggested withdrawal of the antiserum–wet virus product.^139^

We would emphasise that warning tonight, for the amount of irritation caused to the dog owner, and veterinary surgeons, and the amount of troublesome correspondence, visits to packs, etc., which are imposed upon our staff are, in our opinion, too great a price to pay for the use of this method in its present form.^140^

In the event a solution was soon forthcoming, but not from the WPRL's laboratories or Mill Hill. Instead, it came from some keen veterinarians, working independently with the method in the field. They found that rather than injecting the virus and antiserum simultaneously, as recommended, much better results were produced by first injecting the virus and then waiting a few hours to inject the serum; the interval appeared to reduce failures and make immunity more durable.^141^ Dalling and his colleagues tested and approved the veterinarians’ method, and, as it was increasingly adopted, complaints about serum–virus prophylaxis moderated. Within a year, BWC was marketing its vaccine–virus and serum–virus as the leading products for distemper control.^142^

## Conclusion

The two WPRL products were such a success that at the end of 1932 the FDF was wound up and its achievements celebrated in the special supplement of *The Field* in February 1933.^143^ The Distemper Research Council set out a simple narrative of how the laboratory research that it had funded had been rapidly translated into two effective vaccines that would ‘save the lives of our dogs’. The MRC was also in self-congratulatory mood. Henry Dale described it as an exemplar of ‘a complete and systematic investigation of a virus disease’, and its culmination in the large-scale production of a vaccine symbolized the importance of the NIMR to the nation.^144^ Indeed, the work was said to be important for the empire, promising to save the silver fox fur trade in Canada.^145^

Laidlaw and Dunkin received all the public plaudits, though the FDF's final report did acknowledge the role of commercial laboratories making ‘their results available to the veterinary profession and so to the public for the benefit of every dog’.^146^ However, the view at the WPRL was that their work had not been adequately recognized:

While the patient and brilliant experimental work of Laidlaw and Dunkin has been duly acclaimed, it is nevertheless desirable that tribute should be made to the associated workers of WPRL who have been able to translate laboratory experimental work into commercial scale production. It is, perhaps, almost unnecessary to state that many technical questions regarding the preparation, standardisation and suitability had to be investigated and solved.^147^

We would concur with this assessment, but would add to those deserving credit the constituencies who raised and made voluntary donations, and the many veterinarians, masters of hounds and dog owners who volunteered to conduct field trials and feed back results. Our narrative of the distemper project is telling evidence against the linear model of innovation that still dominates the rhetoric, and perhaps much current policy thinking, on translational research.

The NIMR's work on distemper helped build capacity for the MRC at Mill Hill and was the foundation for its work on virus diseases for the next decade. It demonstrated the redundancy of any strict human–animal boundary in medical research. Already in 1930, the MRC devoted an entire volume to ‘Viruses and virus diseases’ in its definitive *System of Bacteriology in Relation to Medicine* and Laidlaw contributed a chapter on dog distemper, which took its place alongside smallpox, mumps, measles, yellow fever, poliomyelitis and a range of animal, plant, insect and bacterial virus diseases.^148^ In 1931, Fletcher observed that

it is already clear that the usefulness of this work is not to be limited to the prevention and cure of canine distemper. In the field of medical research the work has at many points aided the development of technical methods for the study of viruses in general.^149^

The ferret became the model animal for the NIMR's next big research programme on human influenza. Their two-stage vaccine–virus method of immunization was incorporated into the development of vaccines being recommended for poliomyelitis, yellow fever and rinderpest.^150^ Significant in the longer term were the range of immunization methods developed and trialled, and the demonstration of the need to make protocols workable technically and socially.

What makes this campaign novel was how its translational and scientific characteristics were intimately linked to the ethos underpinning its organization. The fund was rooted in a kind of voluntarism that had been cultivated by Victorian aristocrats and *noblesse oblige* continued to shape patrician values. At its core was the idea that propertied elites had a duty to the nation, which could take such forms as participating in politics or government administration, ministering to the poor, or providing financial support for various causes. In the case of dog distemper, this ethos went well beyond monetary contributions, as members of the FDC played an active role in its administration and direction. In the twentieth century, the landed classes have often been portrayed as indifferent, if not hostile, to modern science and technology, preferring to look backwards rather than forwards.^151^ The role that key individuals played in the distemper research programme certainly aided the growth and advanced the profile of biomedical science at many levels, though it has to be said that a key reason for their support was conservative, to preserve foxhunting. While landed patricians were the leaders of the enterprise, they drew into the scheme dog owners from all social classes, from across the country, the empire and the world. Not only did individuals send in donations, but they also followed the progress of the research in popular publications, corresponded with NIMR and WPRL researchers, volunteered pets for trials, and, after 1931, were willing to pay to have their dogs immunized by one of the two methods available. Here was a precedent, not just for public engagement with the results of research, but in all stages of the research process and in the translations of inventions into useful innovations. We suspect that this pattern of patronage and engagement was common in interwar British medical and veterinary scientific research, and thus worthy of further historical attention.

And dogs played a vital role, too, and their agency should also be recognized. The common rallying point for the distemper campaign was the plight of the nation's dogs and they had multiple and changing roles. They were experimental bodies for the developing, testing and mass-production of tools for their protection. Different dogs served different purposes in this process. A new laboratory dog was specially created for the development and commercialization of the distemper vaccine. Cross-bred rather than pedigree, this dog underpinned the manufacture of a vaccine for all breeds. Trials of the vaccine relied on different breeds, in packs, kennels, and homes, which served as populations living in ‘natural’ or ‘field’ conditions. Dogs were also ‘factories’ for the large-scale production of vaccines and antisera, and used as test vehicles for the standardization of the products. However, dogs’ variable constitutional susceptibility to distemper and their sociability meant that they were ill-suited to this form of laboratory life, and thus for crucial aspects of the research they had to be replaced by ferrets, which became essential experimental animals. The outpouring of public good will towards and support for the campaign was rooted in the status of dogs as valued companions, from high-bred members of hunting packs to mongrel family pets. Because their dogs were so crucial, the stakeholders enrolled in the campaign demanded levels and types of engagement not previously seen in research and development in Britain, and high standards of safety and efficacy from its results. Only by paying attention to the many roles of animals has it been possible to make visible and analyse this crucial aspect of British interwar science and medicine.

## Footnotes

1 Frederick Hobday, ‘Saving the lives of our dogs’, The Field, 4 February 1933, p. 1.

2 Victor Horsley, ‘Rabies in dogs’, The Standard, 29 May 1889, p. 5.

3 E.M. Tansey, ‘Protection against dog distemper and Dogs Protection Bills: the Medical Research Council and anti-vivisectionist protest, 1911–1933’, Medical History (1994) 38, pp. 1–26814560610.1017/s0025727300056027PMC1036808; Joan Austoker and Linda Bryder, ‘The National Institute for Medical Research and related activities of the MRC’, in Austoker and Bryder (eds.), Historical Perspectives on the Role of the MRC: Essays in the History of the MRC of the United Kingdom and Its Predecessor, the Medical Research Committee, 1913–1953, Oxford: Oxford University Press, 1989, pp. 40 and 43. The ‘official’ history of the council describes the work at the NIMR and unsurprisingly says little on its context. See A. Landsborough Thomson, Half a Century of Medical Research: The Programme of the Medical Research Council (UK), vol. 2, London: HMSO, 1975, pp. 119–122. The commercial story of the vaccine at Burroughs Wellcome, with little reference to the MRC, is told in R.A. Church and E.M. Tansey, Burroughs, Wellcome & Co.: Knowledge, Trust and Profit, and the Transformation of the British Pharmaceutical Industry, Lancaster: Carnegie Publishing, 2007, pp. 349–351.

4 The products available in 1933 were a vaccine containing dry, killed distemper virus; live virus used to reinforce vaccination; distemper antiserum used to treat infected dogs; and live virus to vaccinate healthy dogs.

5 Roy M. Macleod and E.K. Andrews, ‘The origins of the DSIR: reflections on ideas and men, 1915–1916’, Public Administration (1970) 48, pp. 23–48; Peter Alter, The Reluctant Patron: Science and the State in Britain 1850–1920, Oxford: Berg, 1987; Joan Austoker, ‘Walter Morley Fletcher and the origins of a basic biomedical research policy’, in Austoker and Bryder, *Historical Perspectives*, op. cit. (3), pp. 22–33; David Edgerton and Sally M. Horrocks, ‘British industrial research and development before 1945’, Economic History Review (1994) 47, pp. 213–238; David Edgerton, Science, Technology and the British Industrial ‘Decline’, 1870–1970, Cambridge: Cambridge University Press, 1996; Edgerton, Warfare State: Britain, 1920–1970, Cambridge: Cambridge University Press, 2005; Sabine Clarke, ‘Pure science with a practical aim: the meanings of fundamental research in Britain, circa 1916–1950’, Isis (2010) 10, pp. 285–3112071827310.1086/653094.

6 Donald Fisher, ‘Rockefeller philanthropy and the British Empire: the creation of the London School of Hygiene and Tropical Medicine’, History of Education (1978) 7, pp. 129–1431161457010.1080/0046760780070206; Fisher, ‘The Rockefeller Foundation and the development of scientific medicine in Great Britain’, Minerva (1978) 16, pp. 20–411161066210.1007/BF01102180; Christopher Lawrence, Rockefeller Money, the Laboratory, and Medicine in Edinburgh, 1919–1930: New Science in an Old Country, Rochester, NY: University of Rochester Press, 2005.

7 For the interests of landed elites we have relied on David Cannadine, The Decline and Fall of the British Aristocracy, New Haven: Yale University Press, 1990. For their relative position and role in British society see Jose Harris, Private Lives, Public Spirit: A Social History of Britain, 1870–1914, Oxford, Oxford University Press, 1993. The history of the British veterinary profession has received limited attention, see Iain Pattison, The British Veterinary Profession, London: J.A. Allen, 1984.

8 Emma Griffin, Blood Sport: Hunting in Britain since 1066, New Haven: Yale University Press, 2008; J. Blancou, ‘Dog distemper: imported into Europe from South America?’, Historia medicinae veterinaria (2004) 29, pp. 35–4115376360.

9 Pamela Hunter, Veterinary Medicine: A Guide to the Sources, Aldershot: Ashgate, 2004, p. 11. The first modern dog show was held in Newcastle upon Tyne in July 1859. The Kennel Club was established in 1873.

10 William Hunting, ‘Vaccination against distemper’, Veterinary Record (1902) 14, p. 373.

11 Andrew Gardiner, ‘“The loathsome complaint”: the early history of canine distemper’, Veterinary History (2008) 14, pp. 105–114.

12 J.R. Fisher, ‘Not quite a profession: the aspirations of veterinary surgeons in England in the mid-nineteenth century’, Historical Research (1993) 66, pp. 284–302; Abigail Woods and Stephen Matthews, ‘“Little, if at all, removed from the illiterate farrier or cow-leech”: the English veterinary surgeon, *c*.1860–1885, and the campaign for veterinary reform’, Medical History (2010) 54, pp. 29–542004626310.1017/s0025727300004300PMC2793141; Anne Hardy, ‘Professional advantage and public health: British veterinarians and State Veterinary Services, 1865–1939’, 20th Century British History (2003) 14, pp. 1–231268829510.1093/tcbh/14.1.1. Antje Grünberg, ‘The distemper of the dog: a contribution to the history of pet diseases’, PhD dissertation, University of Berlin, 1997.

13 Hunter, op. cit. (9), p. 11.

14 Hamilton Kirk, Canine Distemper: Its Complications, Sequelae and Treatment, London: Baillière, Tindall & Cox, 1922.

15 ‘W’, ‘The distemper in dogs’, *New Sporting Magazine* (1847) 13, p. 439. For similar views see, for example, Mr McBride, ‘On ulcers of the cornea in dogs suffering from distemper’, British Medical Journal (12 February 1870) 1, p. 159; George Fleming, A Manual of Veterinary Sanitary Science and Police, London: Chapman & Hall, 1875, pp. 290–291.

16 Michael Worboys, ‘Germ theories of disease and British veterinary medicine, 1860–1890’, Medical History (1991) 35, pp. 308–327188643010.1017/s0025727300053813PMC1036483; Abigail Woods, A Manufactured Plague? The History of Foot and Mouth Disease in Britain, London: Earthscan, 2004, pp. 1–19.

17 On early distemper vaccines see Ian Tizard and Roland D. Schultz, ‘Grease, anthraxgate, and kennel cough: a revisionist history of early veterinary vaccines’, Advances in Veterinary Medicine (1999) 41, pp. 7–24989000610.1016/s0065-3519(99)80005-6.

18 Everett Millais, ‘The pathogenic microbe of distemper in dogs, and its use for protective inoculation’, British Medical Journal (1890) 1, pp. 856–85910.1136/bmj.1.1528.856-aPMC220748320753012.

19 Sidney Monckton Copeman, ‘The micro-organism of distemper in the dog and the production of a distemper vaccine’, Proceedings of the Royal Society of London (1900) 67, pp. 459–461.

20 Charles Phisalix, ‘Maladie des jeunes chiens: Statistique des vaccinations pratiquées du 15 mai au 15 août 1902’, Comptes rendus de l'Académie des sciences (1902) 134, 1252. Lignières and Phisalix's bacillus is discussed in Kirk, op. cit. (14), pp. 32–33.

21 ‘Report of a Committee formed to carry out experiments with the vaccine of Dr. Phisalix’, *Journal of Comparative Pathology and Therapy* (1904) 17, p. 274; ‘Some remarks on distemper’, *Veterinary* Record (1906) 18, p. 757.

22 Newell S. Ferry, ‘Etiology of canine distemper’, Journal of Infectious Diseases (1911) 4, pp. 399–420; J.P. M'Gowan, ‘Some observations on a laboratory epidemic, principally among dogs and cats, in which the animals affected presented symptoms of the disease called “distemper”’, Journal of Pathology and Bacteriology (1911) 15, pp. 372 ff.

23 ‘Committee on Distemper in Dogs’, *Veterinary Record* (1907) 20, pp. 17–18.

24 Masters of the Puckeridge Hunt were usually bankers or brewers, of whom the Barclays were leading figures. Raymond Carr, English Fox Hunting: A History, London: Weidenfeld and Nicolson, 1976, pp. 81–82. Michael F. Berry, A History of the Puckeridge Hunt, London: Country Life Books, 1950. Obituary, ‘Lord Leconfield: a life of public service’, *The Times*, 18 April 1952, p. 8E; obituary, ‘Major M.E. Barclay: Hunting and Farming’, *The Times*, 16 November 1962, p. 15C; ‘The Puckeridge Hunt, 1725–1946’, Hertfordshire Countryside, available at www.hertfordshire-genealogy.co.uk/data/places/places-p/puckeridge_/puckeridge-foxhounds.htm, accessed 15 May 2012.

25 On the problem of distemper amongst hounds before the First World War see C. Bradley, The Foxhound of the Twentieth Century: The Breeding and Work of the Kennels of England, London: George Routledge and Sons, 1914.

26 Abigail Woods, ‘“Partnership” in action: contagious abortion and the governance of livestock disease in Britain, 1885–1921’, Minerva (2009) 47, pp. 195–216.

27 Keir Waddington, The Bovine Scourge: Meat, Tuberculosis and Public Health, 1850–1914, Woodbridge: Boydell Press, 2006.

28 Report, ‘The Commission on Grouse Disease’, *The Times*, 13 August 1904, p. 4E.

29 ‘Committee on Grouse Disease’, *The Times*, 4 February 1907, p.14B; Louis Cobbett and G.S. Graham-Smith, ‘An investigation of the pathology of “grouse disease”’, Journal of Hygiene10 (1910), pp. 1–362047440710.1017/s0022172400033775PMC2167212.

30 *Nature* (26 October 1911) 87, pp. 544–545.

31 Kirk, op. cit. (14), pp. 58–81.

32 Kirk, op. cit. (14), p. ix.

33 Andrew Gardiner, ‘Small animal practice in British veterinary medicine, 1920–1956’, unpublished PhD thesis, University of Manchester, 2009, pp. 112–115.

34 Hardy, op. cit. (12), pp. 8–9; Hunter, op. cit. (9), pp. 23–4; Frederick T.G. Hobday, Surgical Diseases of the Dog and Cat: With Chapters on Anaesthetics and Obstetrics, London: Baillière Tindall and Cox, 1901.

35 Woods, op. cit. (16), pp. 68–81.

36 Hobday, op. cit. (1), p. i.

37 Cook to Fletcher, meeting to discuss ‘The Distemper Question’, 21 October 1922, National Archives, Kew Gardens, London (hereafter NA), FD1/1274.

38 Cook to Fletcher, 22 November 1922, NA FD1/1274.

39 Steve Sturdy, ‘War as experiment: physiology, innovation and administration in Britain, 1914–1917: the case of chemical warfare’, in Roger Cooter, Mark Harrison and Steve Sturdy (eds.), War, Medicine and Modernity, London: Sutton, 1999, pp. 65–84.

40 Jonathan Liebenau, ‘The MRC and the pharmaceutical industry: the model of insulin’, in Austoker and Bryder, *Historical Perspectives*, op. cit. (3), pp. 163–180; Michael Bliss, The Discovery of Insulin, Toronto: University of Toronto Press, 1982.

41 Tansey, op. cit. (3), p. 11; Tansey, ‘The early scientific career of Sir Henry Dale F.R.S. (1875–1968)’, unpublished PhD dissertation, University of London, 1990; Christiane Sinding, ‘Making the unit of insulin: standards, clinical work, and industry, 1920–1925’, Bulletin of the History of Medicine (2002) 76, pp. 231–2771206079010.1353/bhm.2002.0097.

42 Michael Bresalier, ‘Neutralizing flu: “immunological devices” and the making of a virus disease’, in K. Kroker, J. Keelan and P.M.H. Mazumdar (eds.), Crafting Immunity: Working Histories of Clinical Immunology, Aldershot, Ashgate, 2008, pp. 107–144; Bresalier, ‘Uses of a pandemic: forging the identities of influenza and virus research in interwar Britain’, Social History of Medicine (2008) 25, pp. 400–42410.1093/shm/hkr162PMC731377034191895.

43 Fletcher to C.J. Martin, 13 October 1922: ‘It is, I think, quite easy to justify [dog distemper], though we may have some possible political difficulty justifying the expenditure of the Medical Research Fund upon the study of an animal disease.’ NA FD1/1275.

44 Fletcher to Cook, 14 November 1922, NA FD1/1274.

45 Woods, op. cit. (16), pp. 76–7.

46 See Advisory Committee on research into diseases in animals, NA FD 1/4364.

47 Robert E. Kohler, ‘Walter Fletcher, F.G. Hopkins, and the Dunn Institute of Biochemistry: a case study in the patronage of science’, Isis (1978) 69, pp. 331–35538765910.1086/352064; Lawrence, op. cit. (6), pp. 11–23; Robert E. Kohler, Partners in Science: Foundations and Natural scientists, 1900–1945, Chicago: The University of Chicago Press, 1991.

48 ‘Research on distemper’, *The Lancet* (1924) 1, p. 1010. Details of contributions were published in Hobday, op. cit. (1).

49 Susan D. Jones, Valuing Animals: Veterinarians and Their Patients in Modern America, Baltimore: Johns Hopkins University Press, 2003, p. 132; A. Eichhorn, ‘Credit where credit is due (letter to the editor)’, Journal of the American Veterinary Medical Association (1934) 85, pp. 823–824. The American Distemper Committee found it harder to raise money, its scientists continued to work on ‘bacteria’ rather than viruses, and thus it continually deferred to the FDC and the NIMR. See *New York Times*, 22 April 1923, p. 156; 27 January 1929, p. S6; 5 May 1929, p. 168; 7 July 1929.

50 ‘The cure and causes of distemper’, the *Field* Distemper Council, November 1924, NA FD/1275.

51 The council's vice-president, the 6th Duke of Portland, William Cavendish-Bentinck, was an Old Etonian and a Conservative peer who had served as Master of the Horse. He ran the family estate and stud farm in Nottinghamshire made famous by Lord Henry Bentinck, who was master of the Burton Foxhunt (1842–1862), whose notes on *Foxhounds and Their Handling in the Field* were compiled by his son, Lord Charles Bentinck, also a council member. The chairman was Lord Goschen, 2nd Viscount of Hawkhurst, Kent, a Conservative MP and member of the Privy Council, who previously served as governor of New South Wales and secretary to the Board of Agriculture and Fisheries. When he stepped down in 1924 to become governor of Madras, and later Viceroy of India, his position was taken by George Lane Fox, the 1st Baron of Bingley, graduate of Eton and New College, Oxford, a Conservative MP and member of the Privy Council, whose grandfather – ‘The Squire’ of Bramham Park – was master of the Bramham Moor Hunt on the family estate. The council's treasurer was Lord Mildmay of Flete. Educated at Eton and Trinity College, Cambridge, he was a Liberal then Conservative MP, and an original member of the MRC. He received a baronage in 1922 and lived in Flete House, a mansion near Plymouth, where he bred and exhibited South Devon cattle. Overall, one-half of the thirty British members of the Distemper Council retained peerages and were from the landed elite. There were four dukes, three earls, two viscounts, six lords, and a baron. All had direct links to hunts or country sports. In addition, the council included Edward E. Barclay; Arthur Henry Holland Hibbert, whose kennel at Munden House bred the finest strains of Labrador retriever; and Francis Redmond, president of Kennel Club.

52 Members from the medical and veterinary professions included (Sir) Humphry Rolleston, president of the Royal College of Physicians; (Sir) Ray E. Lankester, former director of the Natural History Museum; (Sir) David Bruce, chairman of the Lister Institute; (Sir) Frederick Hobday; Professor A.J. Sewell, canine surgeon to the King and Kennel Club; Harold Leeney, MRCVS, veterinary surgeon and author of *The Animal Doctor* (1913) and *Home Doctoring of Animals* (1929); and Sir William Boog Leishman, expert in tropical diseases, a founding member of the MRC, and adviser on pathology to the War Office.

53 Maisie Fletcher, The Bright Countenance: A Personal Biography of Walter Morley Fletcher, London: Hodder and Stoughton, 1957; T.R.E., ‘Sir Walter Morley Fletcher, 1873–1933’, *Obituary Notices of Fellows of the Royal Society* (December 1933) 1, pp. 153–163.

54 Letter to Lord Astor from Lord Mildmay, 1 December 1932, *Field* Distemper Council, NA FD1/1281. Also see Hobday, op. cit. (1), p. iii.

55 Figures based on reports issued by the *Field* Distemper Fund, NA FD 1/1274. Report for January 1927, ‘Details of Contributions to *The Field* Distemper Fund to the end of 1925’, pp. 12–18; ‘Details of Contributions to *The Field* Distemper Fund for 1926’, pp. 18–20. ‘Details of Contributions to *The Field* Distemper Fund for 1927 and to the 3rd of December, 1928’, in P.P. Laidlaw and F.W. Dunkin, ‘A report upon the cause and prevention of dog distemper’, Progress Report of the Distemper Research Committee, the *Field* Distemper Fund, 1928, pp. 19–20.

56 ‘Details of contributions to *The Field* Distemper Fund’, 1930, NA FD1/1296.

57 Henry H. Dale, ‘Patrick Playfair Laidlaw, 1881–1940’, Obituary Notices of Fellows of the Royal Society (1941) 3, pp. 427–447.

58 Thomas Dalling, ‘George William Dunkin, 1886–1942’, Journal of Pathology and Bacteriology (1942) 54, pp. 401–402.

59 Laidlaw and Dunkin, op. cit. (55), pp. 5–6. The need for controlled supplies of animals was identified as a general issue for the NIMR from its creation. See Robert G.W. Kirk, ‘“Wanted – standard guinea pigs”: standardisation and the experimental animal market in Britain ca.1919–1947’, Studies in History and Philosophy of Biological and Biomedical Sciences (2008) 39, pp. 283–28510.1016/j.shpsc.2008.06.002PMC330256418761280.

60 MRC, *Report of the Medical Research Council for the Year 1930–1931*, London: HMSO, 1932, p. 27.

61 ‘Field Distemper Fund’, Fletcher to Cook, 24 January 1924, NA FD1/1275.

62 Laidlaw and Dunkin, op. cit. (55), p. 6.

63 ‘Visit to Mill Hill Farm Laboratories’, *Veterinary Record* (1928) 8, p. 1102.

64 Second Report of the Dog Distemper Research Committee, February 1924.

65 Laidlaw and Dunkin, op. cit. (55), p. 5.

66 Laidlaw and Dunkin, op. cit. (55), p. 5.

67 P.P. Laidlaw and G.W. Dunkin, ‘Studies in dog distemper. V. The immunisation of dogs’, Journal of Comparative Pathology and Therapeutics (1928) 41, pp. 209–227; Laidlaw and Dunkin, op. cit. (55), pp. 13–15.

68 G.W. Dunkin and P.P. Laidlaw, ‘Studies in dog-distemper. II. Experimental distemper in the dog’, Journal of Comparative Pathology and Therapeutics (1926) 39, pp. 213–221.

69 MRC, Report of the Medical Research Council for the Year 1924–1925, London: HMSO, 1926; MRC, Report of the Medical Research Council for the Year 1925–1926, London: HMSO, 1927. For discussion of the general problem of filtration in virus work, see Ton van Helvoort, ‘History of virus research in the twentieth century: the problem of conceptual continuity’, History of Science (1994) 32, pp. 190–19410.1177/00732753940320020411639267.

70 P.P. Laidlaw, and G.W. Dunkin, ‘Studies in dog-distemper. III. The nature of the virus’, Journal of Comparative Pathology and Therapeutics (1926) 39, p. 228.

71 ‘Visit to Mill Hill Farm Laboratories’, *Veterinary Record* (1928) 8, pp. 1101–1103, 1102. Later, their vaccine work demonstrated that, unlike many other pathogenic viruses identified at this time, distemper was not characterized by many strains.

72 Tansey, op. cit. (3), pp. 12–13.

73 Dunkin and Laidlaw, op. cit. (68), p. 213.

74 Third Report of the Distemper Research Committee – Ferrets, 7 October 1924, NA FD1/1275. On ferrets see Arthur R. Harding, Ferret Facts and Fancies: A Book of Practical Instructions on Breeding, Raising, Handling and Selling; Also Their Uses and Fur Value, Columbus: A.R. Harding, 1919.

75 Third Report of the Distemper Research Committee – Ferrets, 7 October 1924. The veterinary surgeons, Henry Gray and A.J. Sewell, who had battled over distemper in the early 1900s, also informed Laidlaw and Dunkin that they had succeeded in infecting ferrets with distemper. G.W. Dunkin and P.P. Laidlaw, ‘Studies in dog-distemper. I. Dog-distemper in the ferret’, Journal of Comparative Pathology and Therapeutics (1926) 39, p. 201–212.

76 Alexander P. Thomson, ‘A history of the ferret’, Journal of the History of Medicine (1951) 6, pp. 471–480; C. Sweet, R.J. Fenton and G.E. Price, ‘The ferret as an animal model of influenza virus infection’, in Oto Zak and Merle A. Sande (eds.), Handbook of Animal Models of Infection: Experimental Models in Antimicrobial Chemotherapy, London: Academic Press, 1999, pp. 288–298.

77 ‘Visit to Mill Hill Farm Laboratories’, op. cit. (71), p. 1101.

78 Laidlaw and Dunkin, op. cit. (55), p. 10.

79 Laidlaw and Dunkin, op. cit. (75), pp. 209–210.

80 The issue was widely explored in late 1920s and early 1930s, with considerable work being done at the NIMR. See, for example, S.P. Bedson, ‘Observations on the mode of action of a viricidal serum’, British Journal of Experimental Pathology (1928) 9, pp. 235–240; C.H. Andrewes, ‘Immunity in virus diseases’, The Lancet (1931) 2, pp. 1046–1049; W.W.C. Topley, An Outline of Immunity, London: Edward Arnold & Co., 1933, pp. 254–273. The problem was also explored at BWC; see R.A. O'Brien, ‘Certain practical aspects of immunity’, British Journal of Medicine (29 November 1927) 2, pp. 975–978.

81 ‘Distemper and influenza’, *The Lancet* (1927) 1, p. 445.

82 Lise Wilkinson, ‘The development of the virus concept as reflected in corpora of studies on individual pathogens. 5. Smallpox and the evolution of ideas on acute (viral) infections’, Medical History (1979) 23, pp. 1–2836846610.1017/s0025727300050997PMC1082396; Wilkinson, ‘The development of the virus concept as reflected in corpora of studies on individual pathogens. 4. Rabies: two millennia of ideas and conjecture on the aetiology of a virus disease’, Medical History (1977) 21, pp. 15–3132040710.1017/s0025727300037145PMC1081892. In interwar Britain, purifying vaccinia virus for the Government Lymph Department was crucial issue tackled by C.H. Ledingham at the Lister Institute. See Harriett Chick, Margaret Hume and Marjorie MacFarlane, War on Disease: A History of the Lister Institute, London: A. Deutsch, 1971, pp. 133–134.

83 Pauline M.H. Mazumdar, ‘“In the silence of the laboratory”: The League of Nations standardizes syphilis tests’, Social History of Medicine (2003) 16, pp. 437–459.

84 P.P. Laidlaw and G.W. Dunkin, ‘Studies in dog distemper. IV. The immunisation of ferrets against dog distemper’, Journal of Comparative Pathology and Therapeutics (1927) 41, pp. 1–17, 5.

85 Laidlaw and Dunkin, op. cit. (84), p. 9–10.

86 Laidlaw and Dunkin, op. cit. (84), p. 3.

87 Laidlaw and Dunkin, op. cit. (84), p. 7–9.

88 Laidlaw and Dunkin, op. cit. (55), pp. 11–12.

89 Laidlaw and Dunkin, op. cit. (55), p. 12.

90 ‘Dr. Laidlaw and Mr. Dunkin on their distemper investigations’, *Veterinary Record* (1928) 8, p. 1104.

91 Laidlaw and Dunkin, op. cit. (55), pp. 16–17.

92 Experiments on living animals … during the Year 1927, House of Commons Papers, 1928, (109) XIX, p. 830.

93 For the concept of control in MRC clinical trials see Martin Edwards, Control and the Therapeutic Trial: Rhetoric and the Therapeutic Trial in Britain, 1918–48, Amsterdam: Rodopi, 200718271087.

94 MRC, Report of the Medical Research Council for the Year 1927–1928, London: HMSO, 1929, p. 106.

95 MRC, op. cit. (94).

96 ‘The inoculation for distemper’, *Veterinary Record* (1929) 9, pp. 123–124. Reprinted from ‘The “Field” Distemper Fund’, *The Field* (January 1929).

97 ‘The prevention of distemper: discovery of an immunity vaccine’, *Veterinary Journal* (1928) 84, pp. 595–596; Laidlaw and Dunkin, op. cit. (55), pp. 596–599.

98 ‘Distemper conquered – Fruit of 5 Years' Work’, *Daily Mirror*, 30 November 1928, p. 1; *Manchester Guardian*, 30 November 1930, p. 20. Also see *Daily Mirror*, 20 October 1928, p. 5A and 30 October 1928, p. 1, including front-page photographs of Laidlaw and Dunkin; *The Times*, 23 December 1926, p. 12 ff.; 25 January 1927, p. 9D; 29 November 1928, p. 9a and Editorial p. 15C; Editorial, 20 December 1929, p. 8G. *The Field*, 30 November 1929 and 21 December 1929.

99 Editorial, ‘Animal immunity’, *Veterinary Journal* (1928) 8, p. 591.

100 Editorial, ‘Distemper research’, *Veterinary Record* (1928) 8, p. 1095.

101 Laidlaw and Dunkin, op. cit. (55), p. 11.

102 Correspondence between the *Field* Distemper Council and BWC, NA FD1/1296.

103 Peter J. Bowler, Science for All: The Popularization of Science in Early Twentieth-Century Britain, Chicago: The University of Chicago Press, 2009.

104 Laidlaw and Dunkin, op. cit. (55), p. 11.

105 Geo. E. Pearson (deputy director, BWC) to E.S. Grew (secretary, *Field* Distemper Council) on monopoly, production, distribution, naming of vaccine, 11 December 1928, NA FD1/1296. Also see Church and Tansey, op. cit. (3), pp. 349–350; H.J. Parish, *The Wellcome Research Laboratories and Immunisation: A Historical Survey and Personal Memoir* – chronologies and biographical notes, mimeograph, *c.*1970, Wellcome Library Archives, WF/M/H/08/19.

106 Progress Report of the Distemper Research Committee, 1929, p. 1, NA FD1/1279.

107 ‘Obituary: Sir Thomas Dalling’, *Times*, 26 May 1982, p. 12G–H.

108 The American companies were Lederle Laboratories and Mulford Laboratories. ‘Success of Vaccine Treatment’, *Veterinary Record* (1930) 10, p. 38; MRC, op. cit. (94), p. 34; Lederle Laboratories, ‘The control of canine distemper’, 1952, p. 4, available at http://babel.hathitrust.org/cgi/pt?id=coo.31924000254262, accessed 10 May 2012.

109 Correspondence between BWC and Field Distemper Council, December 1928–March 1929, NA FD1/1296.

110 ‘Wellcome’ Brand Canine Distemper Prophylactic’, undated; Pearson to Landsborough Thomson (deputy secretary, MRC), 13 February 1929, NA FD1/1296. Veterinary surgeons were issuing the vaccine in sealed phials containing one dose and rubber-capped bottles containing ten doses; liquid virus was issued separately.

111 Thomson to Mildmay, 12 March 1929: ‘Burroughs Wellcome & Co. will be supplying veterinary practitioners on a commercial basis almost immediately’; ‘Confidential to veterinary surgeons’, undated; BWC to Landsborough Thomson, 6 March 1929, NA FD1/1296. Thomas Dalling, ‘Canine distemper: some sequelae to active immunisation’, Veterinary Record (1929) 9, pp. 774–776; Dalling, ‘Canine distemper prophylaxis’ Veterinary Record (1929) 9, pp. 1049–1052.

112 Thomas Dalling, ‘Canine distemper prophylaxis’, Veterinary Record (1929) 9, p. 1049.

113 Dalling, op. cit. (112), p. 1049.

114 Dalling to Miss Olga B. Woodward, MRCVS, Walpole House, Burton-on-Trent, 31 December 1929; Miss Olga B. Woodward, MRCVS, to Lady Burton, 2 January 1930, NA FD1/1279.

115 Holland-Hibbert to Portland, 28 January 1930; Fletcher to Mildmay, 1 February 1930; Fletcher to Portland, 12 February 1930. For Lady Howe's kennel and dogs see www.lorkenfarms.com/banchory_bolo.htm, accessed 14 February 2013, NA FD1/1279.

116 ‘Report of the Vaccination Committee’, *British Medical Journal* (1928) 2, p. 266–268; (1929) 2, pp. 30–1; (1931) 2, p. 478; Major Greenwood, ‘The vaccination problem’, British Medical Journal (1930) 1, pp. 398–40120775302PMC2312843.

117 A.A. Comerford, ‘Two years’ field experience with the preventative treatment of distemper as advocated by Laidlaw and Dunkin’, Veterinary Record (1929) 9, p. 84.

118 Press cutting on ‘Canine hysteria’, WF/M/H/02/24.

119 The inquiry was closely followed by the *Field* Distemper Council, which had its Scientific Committee analyse the problems with the vaccine. ‘Inquiry into experience with the commercial preparation’, Report of *The Field* Distemper Committee, March 1930, NA FD1/1279.

120 Dalling first publicly outlined the issues at a meeting of the Section of Comparative Medicine of the Royal Society of Medicine on 26 February 1930, ‘Discussion on canine distemper’, *Proceedings of the Royal Society of Medicine* (1930) 23, pp. 747–756; reprinted as ‘Canine distemper’, *Veterinary Record* (1930) 10, pp. 467–475.

121 Thomas Dalling, ‘Experiences with distemper immunisation’, Veterinary Record (1930) 10, pp. 225–234.

122 P.P. Laidlaw, ‘Dog distemper and immunisation’, Nature (28 December 1929) 124, pp. 991–993; reprinted in the *Veterinary Journal* (January 1930), pp. 48–51. Report of the Distemper Research Committee: Analysis of Complaints, March 1930, pp. 2–5; Interim Reports of the Scientific Committee: November 1929–March 1930, the *Field* Distemper Research Fund, March 1930, NA FD1/1279.

123 R.A. O'Brien, proposed letter to *Veterinary Record*, 12 July 1930, WF/WBSR/06/09. Also see O'Brien's comments in T. Dalling, ‘Experiences with distemper immunisation’, Veterinary Record (1930) 10, pp. 233–234.

124 O'Brien to Laidlaw, 13 July 1930, WF/WBSR/06/09.

125 R.A. O'Brien to C.M. Wenyon, 14 June 1930, WF/WBSR/06/09.

126 WF/WBSR/06/06–08.

127 See, for example, the discussion section in Dalling, op. cit. (121).

128 Editorial, ‘The “Field” distemper vaccine and virus’, Veterinary Record (1930) 10, p. 541.

129 Quoted in Church and Tansey, op. cit. (3), p. 351.

130 Letter, R.A. O'Brien to Wenyon, 14 June 1930, WF/WBSR/06/09.

131 Thomas Dalling, ‘Further field and laboratory observations in connection with canine distemper prophylaxis’, Veterinary Record (1931) 11, pp. 617–618.

132 P.P. Laidlaw and G.W. Dunkin, ‘Studies in dog distemper. VI. Dog distemper anti-serum’, Journal of Comparative Pathology and Therapy (1931) 44, pp. 1–3.

133 P.P. Laidlaw and G.W. Dunkin, ‘Dog distemper antiserum’, Veterinary Record (1931) 11, pp. 359–367.

134 Thomas Dalling, ‘Canine distemper immunisation: use of various prophylactics in hunting packs’, Veterinary Journal (1931) 87, pp. 553–558.

135 Dalling, op. cit. (131), p. 624; FD1/ 1296 ‘WELLCOME’ Anti-Distemper Serum, 1 June 1931.

136 Dalling, op. cit. (131), p. 619.

137 Thomas Dalling, ‘Observations on canine distemper prophylaxis’, Veterinary Record (1932) 12, p. 743.

138 Dalling, op. cit. (137), p. 743.

139 Dalling, op. cit. (137), p. 748.

140 Dalling, op. cit. (137), p. 743.

141 Thomas Dalling, ‘Recent observations on canine distemper immunisation’, Veterinary Record (1934) 14, p. 1350–1351.

142 See Dalling's comments in G.F. Boddie, ‘The clinical aspect of distemper immunisation’, Veterinary Record (1934) 14, p. 513.

143 Hobday, op. cit. (1), pp. i–ii.

144 Hector C. Cameron, ‘Patrick Playfair Laidlaw’, Guy's Hospital Reports (1940–1941) 90, p. 9.

145 ‘The conquest of distemper’, *The Observer*, 4 December 1932, p. 8F.

146 Quoted in *The Times*, 3 December 1932, p. 7A.

147 Dalling Report, January 1933, WF/WSBR/06/17–18.

148 P.P. Laidlaw, ‘Distemper’, in Paul Fildes (eds.), ‘Viruses and virus diseases’, A System of Bacteriology in Relation to Medicine, vol. 7, London: HMSO, 1930, pp. 232–243.

149 MRC, Report of the Medical Research Council for the Year 1931–1932, London: HMSO, 1933, p. 19.

150 MRC, op. cit. (149), p. 20; Daniel Gilfoyle, ‘Veterinary immunology as colonial science: method and quantification in the investigation of horsesickness in South Africa, *c*.1905–1945’, Journal of the History of Medicine and Allied Sciences (2005) 61, pp. 26–651623949710.1093/jhmas/jrj002.

151 See, for example, Christopher Lawrence and Anna K. Mayer (eds.), Regenerating England: Science, Medicine and Culture in Inter-war Britain, Amsterdam: Rodopi, 2000.

